# Review of the ant genus *Technomyrmex* Mayr, 1872 in the Arabian Peninsula (Hymenoptera, Formicidae)

**DOI:** 10.3897/zookeys.780.26272

**Published:** 2018-08-08

**Authors:** Mostafa R. Sharaf, Hathal M. Al Dhafer, Abdulrahman S. Aldawood

**Affiliations:** 1 Plant Protection Department, College of Food and Agriculture Sciences, King Saud University, Riyadh 11451, PO Box 2460, Kingdom of Saudi Arabia King Saud University Riyadh Saudi Arabia

**Keywords:** Afrotropical Region, Arabian Peninsula, Asir Mountains, Dolichoderinae, male, Middle East, Neotype, new record, Palearctic Region

## Abstract

The taxonomy of the dolichoderine ants of the genus *Technomyrmex* Mayr, 1872 is revised for the Arabian Peninsula. Six species are treated, *T.albipes* (F. Smith, 1861), *T.difficilis* Forel, 1892, *T.briani* Sharaf, 2009, *T.vexatus* (Santschi, 1919), *T.montaseri* Sharaf, Collingwood & Aldawood, 2011, and *T.setosus* Collingwood, 1985. The successful tramp species *T.difficilis* Forel, 1892 is recorded for the first time from the Kingdom of Saudi Arabia (KSA) and Yemen, representing new records for the Palearctic Region. *Technomyrmexvexatus* (Santschi, 1919) is a new species record for Yemen. The queen caste of the rare endemic species, *T.briani* Sharaf, 2009 is described for the first time. A neotype for KSA endemic *T.setosus*Collingwood 1985 is designated based on a specimen collected from the type locality, the Asir Mountains, KSA, including new information on habitats and distribution. A male cast of *Technomyrmex*, possibly of *T.setosus*, is also described. An illustrated key based on the worker caste of the Arabian species of *Technomyrmex* is given. New geographical records and a distribution map for the treated species are presented.

## Introduction

The ant genus *Technomyrmex* Mayr, 1872 is a member of the subfamily Dolichoderinae, with 94 valid species and four fossil species worldwide (Bolton 2017). *Technomyrmex* species are distributed throughout Old World tropical and subtropical zones (Brown 2000), the Oriental-Malesian (Bolton 2007), and Neotropical (Fernández and Guerrero 2008) regions. Most species are arboreal or subarboreal (Collingwood 1985, Bolton 2007), but some species nest directly in the ground (Collingwood 1985, Bolton 2007, Sharaf 2009, Fisher and Bolton 2016). The feeding habits of most species include honeydew produced by a wide range of hemipterans, whereas other species are considered generalized foragers (Brown 2000, Bolton 2007).

Bolton (2007) provided a world revision of the genus in which 90 species were recognized including 37 new species described. Subsequently, a synopsis of the New World species was made available by Fernández and Guerrero (2008) including a key to six species. The males of the Malagasy *Technomyrmex* were diagnosed by Yoshimura and Fisher (2011). Recently, the Taiwanese species were revised, recognizing five species including a description of a new species (Yamane et al. 2018).

*Technomyrmex* is one of the incompletely studied ant genera of the Arabian Peninsula. The first treatment of the ants of the Kingdom of Saudi Arabia (KSA) (Collingwood 1985) reported two species, *T.albipes* (F. Smith, 1861) from the Eastern Region, and *T.setosus* Collingwood, 1985 from the southwestern Asir Mountains. In addition, this author mentioned two additional putative species designated as sp. A. and sp. B., and indicating that these taxa may represent undescribed species. In their work on the ant fauna of the Arabian Peninsula, Collingwood and Agosti (1996) briefly treated and keyed the Arabian *Technomyrmex* species and recorded *T.albipes* and *T.setosus* from Yemen. Subsequently, two new species were added to the Arabian Peninsula, *T.briani* Sharaf, 2009 and *T.montaseri* Sharaf, Collingwood & Aldawood, 2011 from the KSA and Oman, respectively (Sharaf 2009, Sharaf et al. 2011). A key to the Arabian species was included in in the latter work. The faunal list of Al Bahah Province (El-Hawagry et al. 2013) recorded *T.briani* and *T.setosus* from various localities in the Al Sarawat Mountains of KSA.

Recent collecting efforts, especially in the southwestern Mountains of KSA by the senior author and the entomology team of King Saud University Museum of Arthropods (KSMA) have resulted in new material for study. Also, several years of field surveys (2009-2017) throughout KSA using different collecting methods (*e.g.* hand collecting, pitfall traps, beating sheets, light traps, etc.), have added material for study, and importantly new information on the distribution of this genus. The study of this new material has allowed us to provide this updated synopsis of the genus for the Arabian Peninsula, providing identification, distribution, and habitat information.

## Materials and methods

Measurements and indices follow Bolton (2007).

**Measurements**

**TL** Total Length: The total outstretched length of the ant from the mandibular apex to the gastral apex.

**EL** Eye Length: The maximum diameter of eyes in profile.

**HL** Head Length: The length of the head capsule excluding the mandibles; measured in full-face view in a straight line from the mid-point of the anterior clypeal margin to the mid-point of the posterior margin.

**HW** Head Width: The maximum width of the head behind the eyes, measured in full-face view.

**SL** Scape Length: The maximum straight-line length of the scape, excluding the basal constriction or neck that occurs just distal of the condylar bulb.

**PW** Pronotal Width: The maximum width of the pronotum in dorsal view.

**WL** Weber’s length of mesosoma: The diagonal length of the mesosoma in profile, from the most anterior point of the pronotum to the posterior basal angle of the metapleuron.

All measurements are expressed in millimetres.

**Indices**

**CI** Cephalic Index: HW divided by HL × 100.

**DTI** Dorsal Thoracic Index: In dorsal view the length from the mid-point of the anterior pronotal margin to the midpoint of the metanotal groove, divided by PW × 100.

**EPI** Eye Position Index: In full-face view the straight-line length (parallel to the long axis of the head) from the most anterior point of the eye to the anterior clypeal margin, divided by the straight-line length from the most posterior point of the eye to the posterior margin × 100.

**OI** Ocular Index: Maximum diameter of eye divided by HW × 100.

**SI** Scape Index: SL divided by HW × 100.

Throughout the text, ‘w’ stands for ‘worker’ or ‘workers’, ‘q’ for ‘queen’, and ‘m’ for ‘male’.

**Abbreviations of museums**

**CASC**California Academy of Sciences Collection, San Francisco, CA, USA.

**KSMA**King Saud University Museum of Arthropods, Riyadh, Kingdom of Saudi Arabia.

**MHNG**Muséum d’Histoire Naturelle, Genève, Switzerland.

**NHMB**Naturhistorisches Museum, Basel, Switzerland.

**OXUM** Hope Entomological Collection, Oxford Museum of Natural History, Oxford, United Kingdom.

**UABC**Universitat Autònoma de Barcelona, Bellaterra, Spain.

**WMLC**World Museum Liverpool, Liverpool, United Kingdom.

**Abbreviations of collecting technique**

**BS** Beating sheet.

**LT** Light trap.

**MT** Malaise trap.

**PT** Pitfall trap.

During more than 20 field trips to the southwestern mountains of KSA, more than 500 specimens were collected using hand picking, pitfall traps, beating sheets, and sifting trays. Beating sheets and sifting trays are efficient methods for collecting this genus of arboreal and ground dwelling ants. All specimens were preserved in 95% ethanol in the field. Ants were later removed and mounted.

## Results

### Diagnosis of the genus *Technomyrmex*

Workers of the genus *Technomyrmex* are distinguished by the following characters (Bolton 2007): Masticatory margin of mandibles armed with 12–14 teeth; palp formula 6, 4; anterior clypeal margin transverse or strongly incised; eyes present; ocelli absent; antennae 12-segmented without a terminal club; metanotal groove well-developed; propodeum unarmed; propodeal dorsum and declivity junction rounded or distinctly angled in profile; petiole reduced, completely concealed by the first gastral tergite when seen from dorsal view; gaster with five tergites visible in dorsal view.

### Synoptic species list of the Arabian *Technomyrmex*

***Technomyrmexalbipes*** (F. Smith, 1861)

= *Technomyrmexnigrum* Mayr, 1872

= *Technomyrmexalbitarse* Emery, 1893

= Technomyrmexalbipesvar.bruneipes Forel, 1895

= Technomyrmexalbipesr.wedda Forel, 1913

***Technomyrmexbriani* Sharaf, 2009**

***Technomyrmexdifficilis* Forel, 1892**

= *Technomyrmexmayrinitidulans* Santschi, 1930

***Technomyrmexmontaseri* Sharaf, Collingwood & Aldawood, 2011**

***Technomyrmexsetosus* Collingwood, 1985**

***Technomyrmexvexatus* (Santschi, 1919)**

### Key to the Arabian *Technomyrmex* Mayr (modified after Bolton 2007, Sharaf et al. 2011)

**Table:** 

1	Head in profile with the dorsal surface of the frontal carina entirely without setae (Fig. 1a); mesosoma without setae (Fig. 1b)	***T.gibbosus*-group**...**2**
–	Head in profile with the dorsal surface of the frontal carina with setae present (Fig. 1c); at least one seta present, or more usually with a row of 2–4; mesosoma with setae (Fig. 1d)	***albipes*-group**...**3**
2	Larger relatively shining brown species (TL 3.0–3.4, HL 0.72–0.78, HW 0.68–0.76, PW 0.44–0.48, WL 0.90–0.96); dorsal outline of mesonotum distinctly convex in profile, with a descending face sloping abruptly back to a deep metanotal groove (Fig. 1e); propodeal dorsum approx. half length of propodeal declivity (Fig. 1e) (Spain, Morocco, and Yemen)	***T.vexatus* Santschi**
–	Smaller dull yellow species (TL 2.2–2.9, HL 0.60–0.62, HW 0.57–0.60, PW 0.37–0.38, WL 0.65–0.80); dorsal outline of mesonotum feebly convex in profile, with a descending face sloping evenly back to a shallow metanotal groove (Fig. 1f); propodeal dorsum approximately one-third length of propodeal declivity (Fig. 1f) (Oman)	***T.montaseri* Sharaf et al.**
3	First gastral tergite usually without setae or rarely with one pair (Fig. 2a) (Saudi Arabia)	***T.briani* Sharaf**
–	First gastral tergite usually with at least seven pairs of setae (Fig. 2b)	**4**
4	Bicolored species, head and gaster brown, mesosoma yellow-brown lighter than head and gaster; setae on first gastral tergite longer with maximum length 0.18 mm (KSA, Oman, Yemen)	***T.setosus* Collingwood**
–	Uniform black-brown to black species; setae on first gastral tergite distinctly shorter with maximum length less than 0.10 mm	**5**
5	Cephalic dorsum behind level of posterior margin of eyes without setae (Fig. 2c); eyes in full-face view fail to break sides of head (Fig. 2d); promesonotum relatively short (DTI 110–124) (Introduced species)	***T.albipes*** (**F. Smith)**
–	Cephalic dorsum behind level of posterior margin of eyes with one or more pairs of setae (Fig. 2e); eyes in full-face view break sides of head (Fig. 2f); promesonotum relatively longer and slender (DTI 127–135) (Introduced species)	***T.difficilis* Forel**

**Figure 1.**
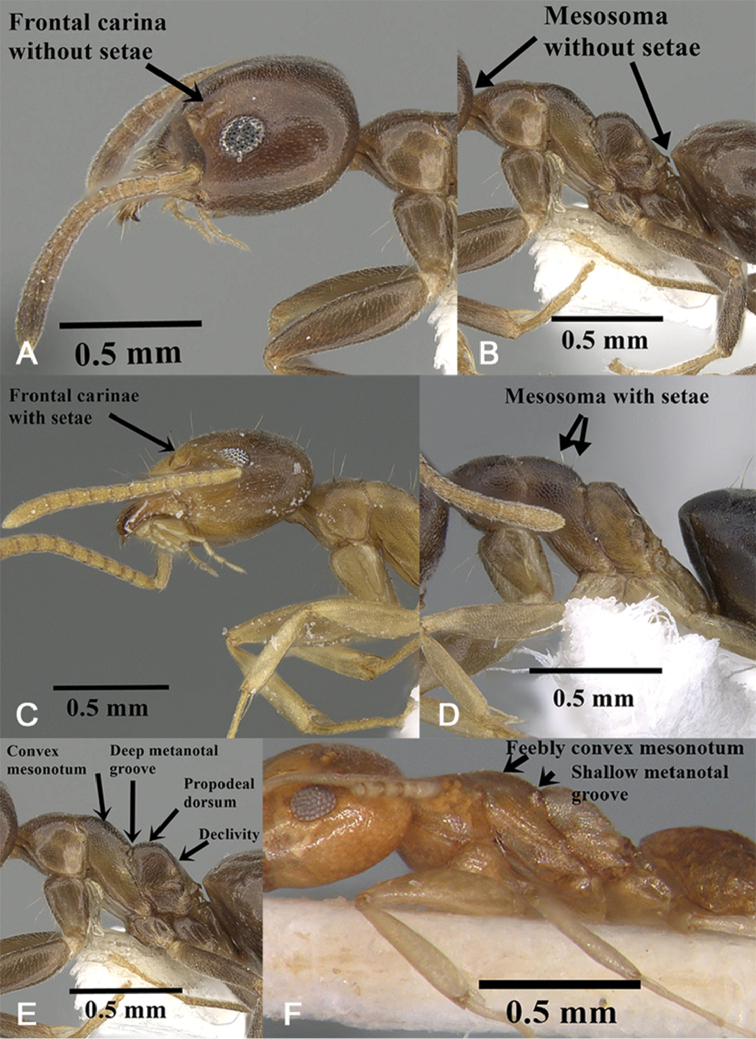
*Technomyrmex* key illustrations: **A***T.vexatus*, head in profile, CASENT0249804**B** mesosoma in profile **C***T.briani*, head in profile, CASENT0919799**D***T.setosus*, mesosoma in profile, CASENT0746639**E***T.vexatus*, mesosoma in profile, CASENT0249804**F***T.montaseri*, mesosoma in profile, images from www.AntWeb.org except **F**.

**Figure 2.**
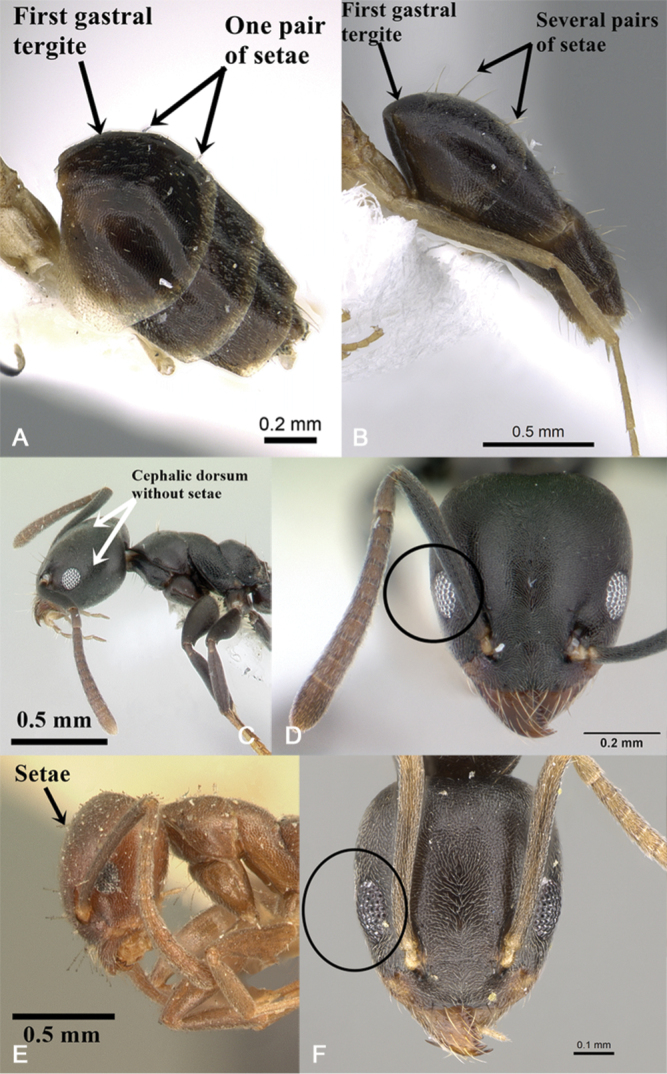
*Technomyrmex* key illustrations: **A***T.briani*, gaster in profile, CASENT0906400**B***T.setosus*, gaster in profile, CASENT0746639**C***T.albipes*, head and mesosoma in profile, CASENT0178469**D***T.albipes*, head in full-face view, CASENT0178469**E***T.difficilis*, head in profile, CASENT0101932**F***T.difficilis*, head in full-face view, CASENT0922887, images from www.AntWeb.org.

### Technomyrmex albipes

Taxon classificationAnimaliaHymenopteraFormicidae

(F. Smith, 1861)

Figure 3A, B, C

Formica (Tapinoma) albipes Smith, 1861: 38 (w.) Syntype worker, Indonesia: Sulawesi, Tond, (A.R. Wallace), CASENT0102952, Indomalaya, (OXUM), (image examined); Forel 1891: 98 (q.); Forel 1908: 21 (ergatoid m.); Karavaiev 1926: 441 (m.); Wheeler and Wheeler 1951: 205 (l.); Crozier 1969: 245 (k.). Combination in Tapinoma: Mayr, 1863: 455; in Technomyrmex: Emery 1888: 392. Senior synonym of Technomyrmexnigrum: Mayr 1872: 147; Mayr 1876: 83; of Technomyrmexalbitarse: Emer 1893: 249; of Technomyrmexbruneipes, Technomyrmexdetorquens, Technomyrmexforticulus, Technomyrmexwedda: Bolton 2007: 68.

#### Description.

**Worker.** Measurements: TL: 2.40–2.90; HL: 0.56–0.63; HW: 0.52–0.58; SL: 0.48–0.58; PW: 0.35–0.42; WL: 0.66–0.78. Indices: CI: 87–95; SI: 91–102; OI: 24–27; EPI: 70–88; DTI: 110–124 (n = 50, from Bolton 2007).

**Head.** Anterior clypeal margin with a feeble, shallow median indentation; head in full-face view with a small shallow indentation medially and strongly convex sides; eyes of moderate size with approximately nine ommatidia in longest row (OI: 24–27), located in front of midlength, with outer margins just fail to break outlines of head sides. **Mesosoma.** In profile the mesonotal outline evenly curved; propodeal dorsum making a distinct obtuse angle with declivity in profile. **Pilosity.** Frontal carina with two pairs of setae; pronotum with 1–3 pairs; mesonotum bare or with one pair (usually none); propodeal dorsum bare; lateral margins of propodeal declivity with one or two pairs, usually with one pair above spiracle, another pair higher up; gastral tergites 1–4 each with abundant scattered long setae (length of setae relatively less than eye diameter or even subequal) on sclerites. **Sculpture.** Body sculpture finely and densely reticulate-punctate, general appearance dull. **Colour.** Head, mesosoma, petiole and gaster black-brown to black; tarsi of mid- and hind legs yellow.

**Figure 3.**
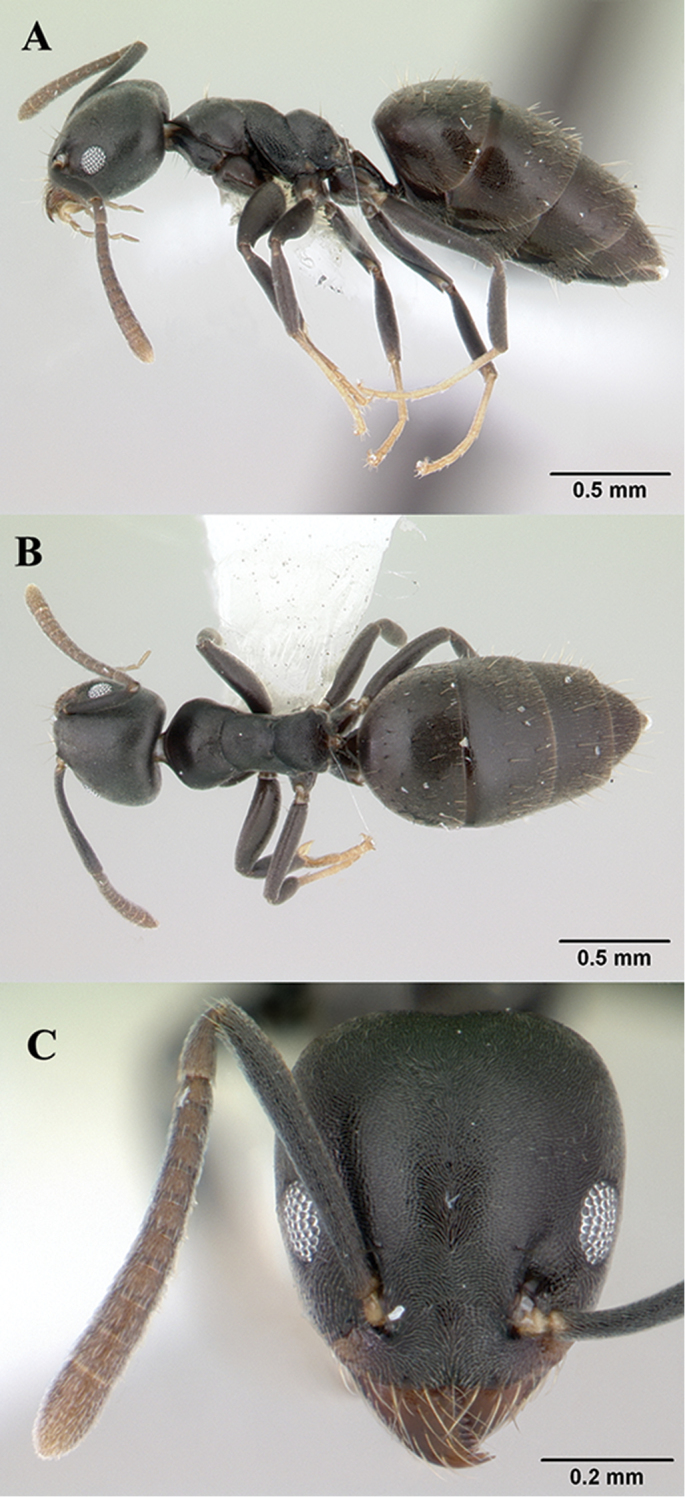
*Technomyrmexalbipes*, worker, CASENT0178469. **A**, body in profile **B** body in dorsal view **C** head in full-face view, images from www.AntWeb.org.

#### Material examined.

KSA, Eastern Province, Hofuf, 25.3142°N, 49.6299°E, 28.v.1978, (W. Büttiker leg.) (1 m) (WMLC).

#### Previous records.

KSA, Eastern Province, Al Qatif, 26.51028°N, 49.96889°E, 30 m, 14.iv.1984, (C. A. Collingwood leg.) (1 m); (Collingwood & van Harten 2001); Yemen: Al Kawd (misspelled AI Kowd), vii.1999, 13.088622°N, 45.364722°E, LT, (Van Harten & Al Haruri leg.); Lahj, iv.1999, 17.1661000°N, 43.3336600°E, MT, (Van Harten & Sallam leg.); Sana’a, 15.3694°N, 44.1910°E, 2250 m, 24.xi.1998, (Van Harten leg.); Ta’iz, V.1998, 13.57952°N, 44.02091°E, LT (Van Harten & Awad leg.) (Collingwood 1985); Al Kadan, vi.2003, 15.248°N, 43.254°E, LT, (Van Harten & T. Abdul Haq leg.) (Collingwood & van Harten 2005).

#### Ecological and biological notes.

*Technomyrmexalbipes* nests and forages in and beneath fallen wood and rocks, in tree trunks, in leaf litter, in twigs, on the forest floor, on low vegetation, and into the canopy (Bolton 2007). The species is known to feed on honeydew of a broad range of sap sucking attended hemipterans including the mealybug vectors of pineapple wilt disease (Sulaiman 1997).

#### Geographic range.

A successful introduced species that has spread worldwide including the Australian, the Afrotropical, and the Malagasy regions (Bolton 2007).

### Technomyrmex briani

Taxon classificationAnimaliaHymenopteraFormicidae

Sharaf, 2009

Figure 4A, B, C

Technomyrmex briani Sharaf, 2009: 213, figs 1–3 (w.), Holotype worker, KSA: Wadi Abha, 18.216389°N, 42.505278°E, 2261 m, 18.iii. 2004, (M. R. Sharaf), 2 paratype workers, same data as the holotype, CASENT0906400, (KSMA), CASENT0911583, (NHMB), (examined), Afrotropic.

#### Description.

**Worker.** Measurements: TL: 2.62–3.0; HL: 0.62–0.72; HW: 0.60–0.67; SL: 0.60–0.72; PW: 0.42–0.47; WL: 0.80–0.92; Indices: CI: 89–100; SI: 92–112; OI: 22–29; EPI: 73–117; DTI: 106–128 (n = 10, from Sharaf 2009).

**Head.** Head distinctly longer than broad with straight posterior margin and clearly curved sides; anterior clypeal margin transverse or very feebly concave medially; posterior margin of head transverse or slightly concave; eyes with 10 ommatidia in the longest row (OI: 22–29) with outer margins just fail to break the outlines of the sides in full-face view; scapes surpass posterior margin of head by approximately ¼ its length. **Mesosoma.** Promesonotal suture distinct; mesonotum in profile evenly rounded descending abruptly to a well-developed metanotal groove; propodeal dorsum short approximately ¼ × length of propodeal declivity. **Pilosity.** Number of setal pairs; frontal carina with two pairs: in profile one pair above the torulus and another pair at the level of the anterior portion of the eye; pronotum with one or two pairs; mesonotum bare or with one or two pairs; sides of propodeal declivity bare or in some individuals with one to three pairs; first, second, and third gastral tergites mostly bare; entire body covered with appressed pale pubescence. **Sculpture.** Body finely superficially granulate, general appearance relatively dull. **Colour.** Bicolored species, head and gaster dark brown, mesosoma yellow-brown clearly lighter than head and gaster; clypeus, mandibles, legs and antennae dirty yellow.

Worker similar to *T.setosus* but it can be separated by the following characters: eyes located relatively posteriorly on head sides; mesosoma and gastral tergites 1–3 mostly bare, rarely promesonotum with one pair of setae.

**Queen.** Measurements: TL: 3.67; HL: 0.80; HW: 0.75; SL: 0.75; PW: 0.62; WL: 1.12; Indices: CI: 94; SI: 100; OI: 29; EPI: 78; DTI: 121. (n = 1).

**Head.** In full-face view with feebly convex sides and nearly straight posterior margin; anterior clypeal margin weak but distinct medially concave; eyes of medium size, with approximately 12 ommatidia in longest row (OI: 29), located on midlength of head, with outer margin of eye touching head sides; scapes when laid back from their insertions surpass the posterior margin of head approximately by the length of first funicular segment. **Mesosoma.** In profile propodeal dorsum and declivity forming a continuous curve; propodeal spiracle located at midlength of declivity. **Pilosity.** Anterior clypeal margin with a single pair of setae; frontal carina with two pairs of black based setae: in profile the first above torulus, the second at about level of anterior margin of eyes, another three setal pairs, behind posterior margin of eyes, in front of small ocelli and on the posterior margin of head. Number of setal pairs on mesosoma: promesonotum and metanotum each with one pair; lateral margins of propodeal declivity with three pairs; first and second gastral tergites each with three pairs on sides, third and fourth tergites each with three pairs scattered on sides. **Sculpture.** Head and mesosoma finely and densely punctate, general appearance dull except gaster feebly shining. **Colour.** Head brown, gaster bark brown, mesosoma yellow-brown, lighter than head, legs and antennae yellow.

**Figure 4.**
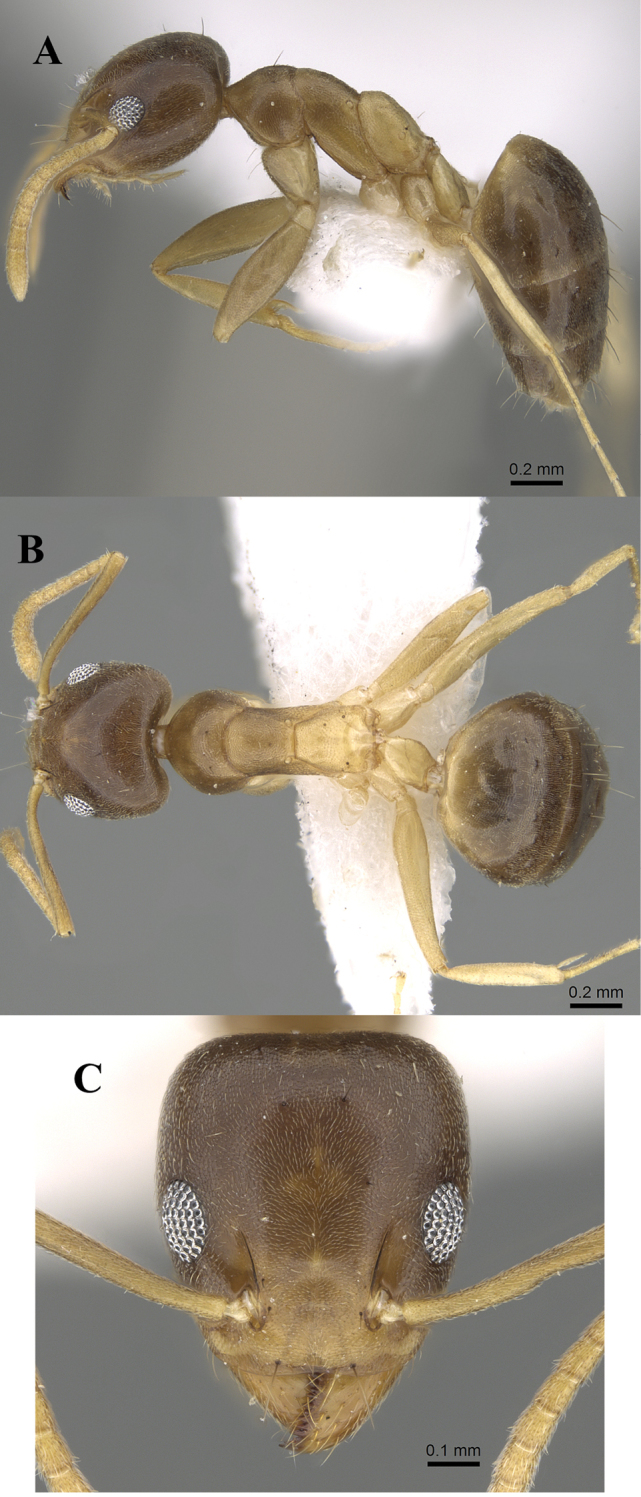
*Technomyrmexbriani*, worker, CASENT0919798. **A** body in profile **B** body in dorsal view **C** head in full-face view, images from www.AntWeb.org.

#### Material examined.

KSA, Al Bahah Province: Elqamh Park, Belgurashi, 19.913056°N, 41.905°E, 1931 m, 17.v.2010, (M. R. Sharaf) (1 w, CASENT0919798, CASC); Shohba Forest, 20.234167°N, 41.623611°E, 2324 m, 14.v.2011, (M. R. Sharaf) (1 w, CASENT0919799, CASC); Shohba Forest, 20.234167°N, 41.623611°E, 2324 m, 14.v.2011, (M. R. Sharaf) (33 w,) ; Saudi Arabia, Al Baha, Wadi Elzaraeb, 20.216944°N, 41.436944°E, 2123 m, 15.v.2010, (M. R. Sharaf) (5 w); Shada Al A’la, 19.838817°N, 41.310067°E, 1563 m, 15.xi.2015, (Al Dhafer et al.), PT, (6 w); Shada Al A’la, 19.8627°N, 41.301483°E, 1225 m, 23.viii.2014, (Al Dhafer et al.), PT, (3 w), all in KSMA.

#### Biological notes.

*Technomyrmexbriani* nests under rocks often next to *Acacia* and *Juniper* trees in southwestern mountains of the KSA. Workers were descending small shrubs and other native plants.

#### Geographic range.

*Technomyrmexbriani* is known only from the KSA and is considered endemic to the southwestern mountains of the Arabian Peninsula.

### Technomyrmex difficilis

Taxon classificationAnimaliaHymenopteraFormicidae

Forel, 1892

Figure 5A, B, C

Technomyrmex mayri r. difficilis Forel, 1892: 242 (w. q.) Syntype worker, Madagascar, Nosibe, Village de l’Imerina coll. (Sikora), CASENT0101932, (MHNG), (image examined), Malagasy. Junior synonym of Technomyrmexmayrinitidulans Santschi, 1930; raised to species and senior synonym of Technomyrmexnitidulans: Bolton 2007: 47.

#### Description.

**Worker.** Measurements: TL: 2.40–3.10; HL: 0.57–0.76; HW: 0.52–0.69; SL: 0.52–0.74; PW: 0.36–0.47; WL: 0.74–1.02; Indices: CI: 89–97; SI: 95–107; OI: 25–30; EPI: 72–86; DTI: 127–135 (n = 35, from Bolton 2007).

**Head.** In full-face view with feebly convex sides and nearly straight posterior margin; anterior clypeal margin weak but distinct medially concave; eyes located in front of midlength of head, with outer margin of eye fail to break head sides. **Mesosoma.** In profile promesonotal and mesonotal outlines forming a continuous curve that descends steeply to a well-defined metanotal groove; propodeal dorsum and declivity meeting in a continuous curve in profile. **Pilosity.** Frontal carina with two pairs of setae: in profile the first above torulus, the second at about level of anterior margin of eyes. Number of setal pairs on mesosoma: pronotum with one or two pairs; mesonotum bare or with one pair; propodeal dorsum bare; lateral margins of propodeal declivity with one or two pairs. Gastral tergites 1–4 each with many pairs of setae, scattered on tergites. **Sculpture.** Body sculpture finely and densely reticulate-punctate, general appearance dull. **Colour.** Head, mesosoma, petiole and gaster dark brown to black; coxae, femora and tibiae of same colour as mesosoma or slightly lighter. Tarsi of middle and hind legs yellow-white to yellow, lighter than tibiae.

#### Material examined.

KSA, Riyadh Province: Riyadh, Almorouj, 24.75837°N, 46.66409°E, 07.x.2017, (M. R. Sharaf) (1 w), KSMA.

#### Ecological and biological notes.

The nesting habit of *T.difficilis* is diverse (Warner 2003). Nests are found in both urban and undisturbed native habitats and are constructed directly into the ground, in trees holes, under palm fronds and old petiole bases, in leaf-litter and under stones and debris. The feeding habits include plant nectar, honeydew, dead insects, and other protein sources. The wide range of nesting sites and feeding habits make *T.difficilis* one of the most successfully dispersed species of the genus worldwide (Bolton 2007). The single specimen studied here was found hiding inside the persistent stamen cluster of a pomegranate fruit imported from Yemen at “Al Othaim Hypermarket”, Riyadh.

#### Geographic range.

*Technomyrmexdifficilis* is broadly distributed worldwide and recorded from the Nearctic (Deyrup 1991), the Australian (Shattuck 1999, Andersen 2000), the Malagasy, the Neotropical, and the Oriental (Bolton 2007) Regions. The present record represents the first for the KSA, Yemen, and the Palearctic Region in general.

**Figure 5.**
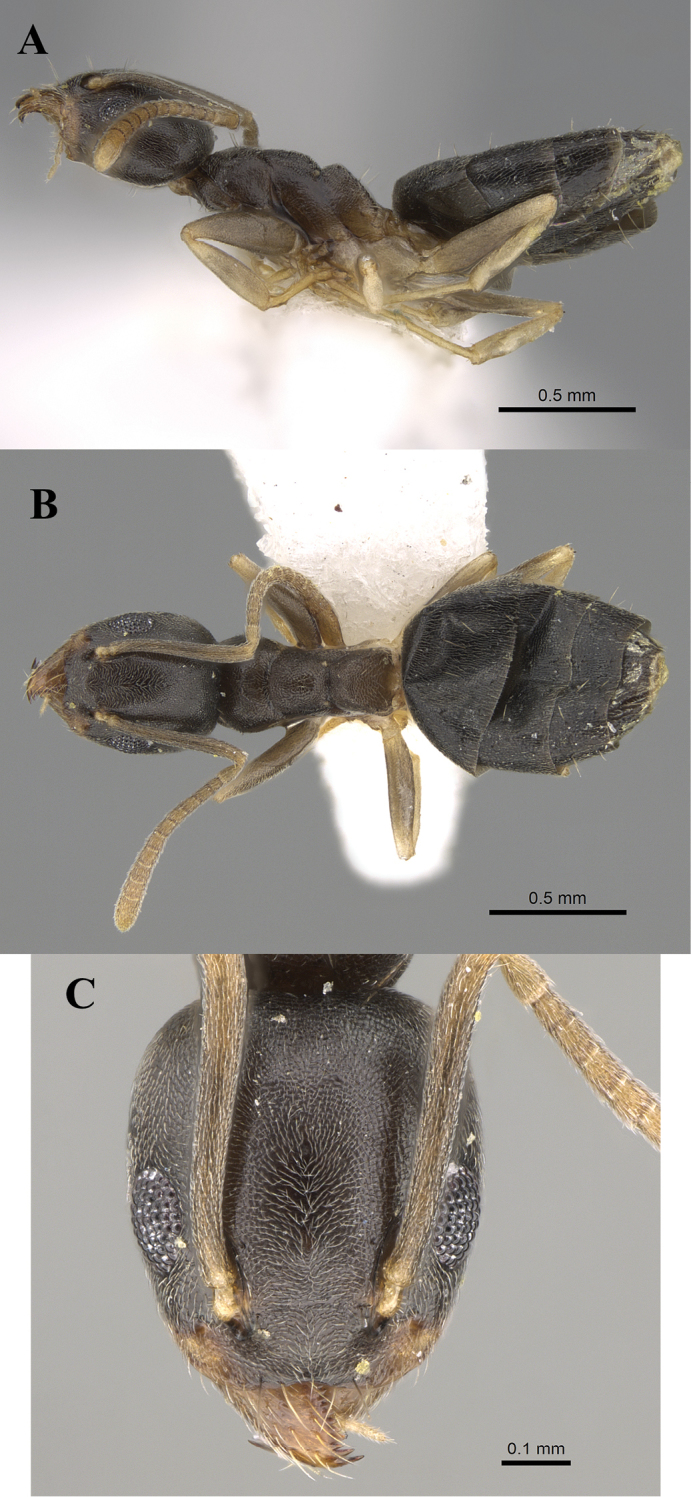
*Technomyrmexdifficilis*, worker, CASENT0922887. **A** body in profile **B** body in dorsal view **C** head in full-face view, images from www.AntWeb.org.

### Technomyrmex montaseri

Taxon classificationAnimaliaHymenopteraFormicidae

Sharaf, Collingwood & Aldawood, 2011

Figure 6A, B, C

Technomyrmex montaseri Sharaf, Collingwood & Aldawood, 2011: 14, figs 1–3 (w.) Holotype worker, Oman: Bani Sur, 24.659°N, 56.494°E 7.iii.1984, (W. Büttiker), (WMLC) (examined), Palearctic.

#### Description.

**Worker.** Measurements: TL: 2.80–2.90; HL: 0.60–0.62; HW: 0.57–0.60; SL: 0.58–0.62; PW: 0.37–0.38; WL: 0.65–0.80; EL: 0.15; Indices: CI: 95–97; SI: 97–109; OI: 25–26; EPI: 80–125; DTI: 122–126 (n = 2). **Head.** In full-face view with feebly convex posterior margin and distinctly convex lateral sides; anterior clypeal margin nearly straight; scapes when laid back from their insertions surpass posterior margin of head by approximately length of first funicular segment; eyes of moderate size with approximately ten ommatidia in longest row (OI: 25–26), located in front of the midlength and their outer margins just failing to break outlines of head sides. **Mesosoma.** In profile mesonotal dorsal outline with short, flat to feebly convex anterior section, posterior section broadly and evenly curved and descending to a deep metanotal groove; propodeum in profile with short convex dorsal surface that rounds evenly into declivity which is nearly three times longer than dorsal face, the two surfaces not separating by an angle. **Pilosity.** Body surface entirely without setae except few long pairs on anterior clypeal margin. **Sculpture.** Body sculpture effaced microreticulum, general appearance more or less dull. **Colour.** Uniformly yellow.

#### Ecological and biological notes.

Nothing is known on ecology or biology of species.

#### Geographic range.

This species is originally described from Oman (Sharaf et al., 2011) and has not been recorded from any other country in the Arabian Peninsula. It is likely endemic to the country.

**Figure 6.**
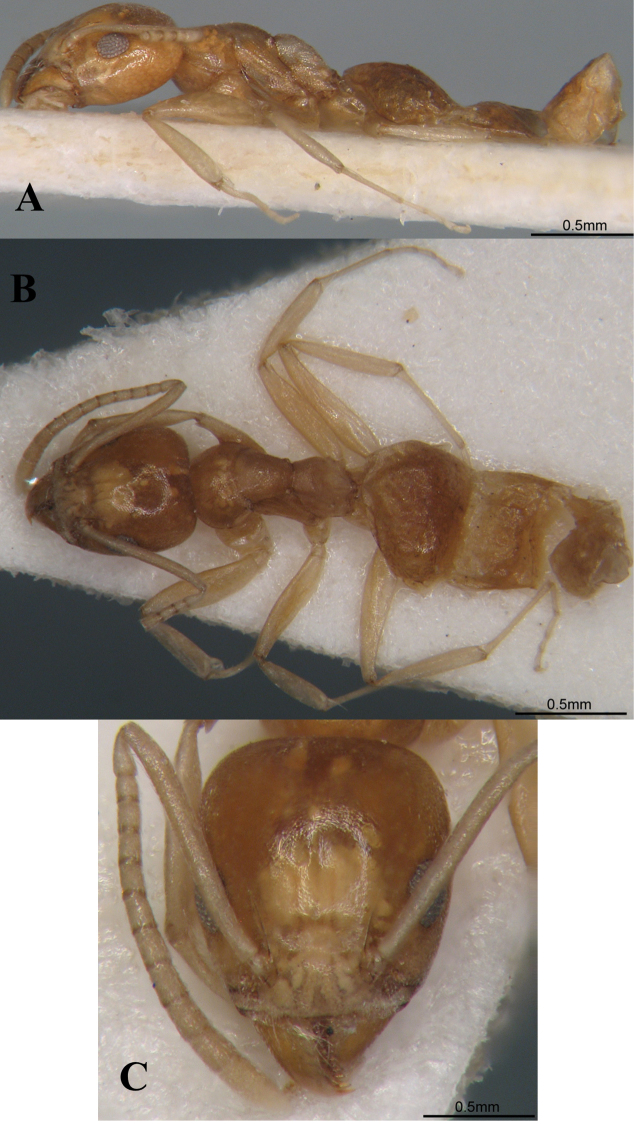
*Technomyrmexmontaseri*, holotype worker. **A** body in profile **B** body in dorsal view **C** head in full-face view.

### Technomyrmex setosus

Taxon classificationAnimaliaHymenopteraFormicidae

Collingwood, 1985

Figure 7A, B, C

Technomyrmex setosus Collingwood, 1985:243, fig. 12. KSA: Wadi Shugub, 7.iv.1983 (C. A. Collingwood) (Holotype worker not in NHMB, presumably lost, Neotype is designated below). Afrotropical.

#### Neotype worker.

KSA, Abha, Alswdah, 18.274167°N, 42.364444°E, 2982 m, 24.iv.2011, (M. R. Sharaf) (CASENT0906357, KSMA) [**here designated].**

#### Description.

**Worker.** Measurements: TL: 2.40–3.27; HL: 0.62–0.67; HW: 0.55–0.62; SL: 0.62–0.70; PW: 0.37–0.45; WL: 0.70–0.80; Indices: CI: 85–100; SI: 105–123; OI: 19–27; EPI: 74–88; DTI: 104–125 (n=9).

**Neotype worker.** Similar to *T.briani* but it can be separated by the following characters: eyes located relatively anteriorly on head sides; first, second and third gastral tergites mostly with abundant scattered pairs of setae.

#### Material examined.

KSA, Shaqiq, 17.71987°N, 42.02869°E, 8.iv.1983 (2 w) (WMLC); **Asir Province**: Gebel Balas (incorrectly as Beles), near Bishah, 19.841389°N, 41.865275°E, 1.ix.1984, (3 w, WMLC); Abha, Raydah Protectorate, 23.iv.2011, 13.221667°N, 42.404167°E, 2600 m, (M. R. Sharaf) (12 w); Abha, Raydah Protectorate, 22.ii.2014, 18.19790°N, 42.40951°E, 2443 m, (M. R. Sharaf), MRS0190, (4 w); Abha, Raydah Protectorate, 28.viii.2014, 18.1961°N, 42.40525°E, 2285 m, (Al Dhafer et al.), PT, (1 w); Abha, Raydah Protectorate, 26.viii.2014, 18.194917°N, 42.4396967°E, 1897 m, (Al Dhafer et al.), PT, (1 w); Abha, Raydah Protectorate, 21.ii.2014, 18.204417°N, 42.4124°E, 2820 m, (M. R. Sharaf), MRS0185, PT, (45 w); Alsawdah, 24.iv.2011, 18.274167°N, 42.364444°E, 2982 m, (M. R. Sharaf) (2 w); Alsawdah, 12.iv.2011, 18.274167°N, 42.364444°E, 2982 m, (M. R. Sharaf) (2 w); **Al Baha Province**: Wadi Turabah, Almandaq, 10.v.2011, 20.310278°N, 41.332222°E, 1793 m, (M. R. Sharaf), BS, (6 w); Shohba Forest, 14.v.2010, 20.234167°N, 41.623611°E, 2324 m, (M. R. Sharaf) (6 w); Wadi Elzaraeb, 20.216944°N, 41.436944°E, 2123 m, 15.v.2010, (M. R. Sharaf) (13 w); Shada Al A’la, 19.842917°N, 41.311517°E, 1666 m, 23.iv.2014, (Al Dhafer et al.), PT, (1 w), all in KSMA.

#### Previous records.

KSA: Gebel Balas (incorrectly written as Beles), near Bishah, Asir Province, 19.841389°N, 41.865275°E, 1.iv.1984; Wadi Al-Farah, Medina, 24.0045°N, 38.005°E, 180 m, 09.viii.1983; Gebel Ghar Harith (written as Harithi), near Najran, 17.479839°N, 44.02525°E, 11.iv.1984 (all collected by W. Büttiker); Yemen: Al-Hajjarah, 15.068889°N, 43.716111°E, 14.iii.1992, (A. van Harten); Oman: no locality (Collingwood & Agosti, 1996).

**Figure 7.**
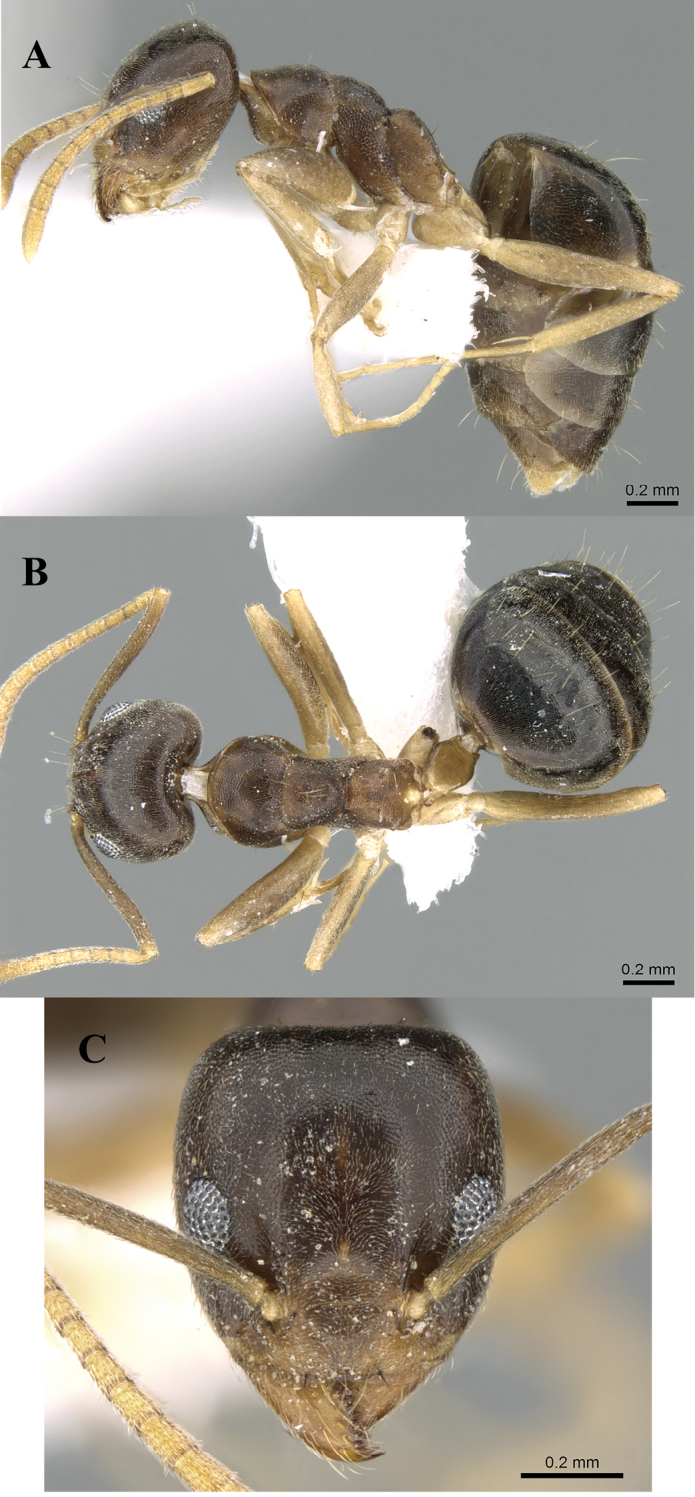
*Technomyrmexsetosus*, neotype worker, CASENT0906357. **A** body in profile **B** body in dorsal view **C** head in full-face view, images from www.AntWeb.org.

#### Remarks.

*Technomyrmexsetosus* was described from the holotype worker and two paratype workers collected from Wadi Shuqub (incorrectly written by Collingwood (1985) as Shugub because of the pronunciation of “q” to “g” by native KSA citizens), Al Bahah Province. The holotype and the two paratypes are not in NHMB and are considered lost. Two workers from Shaqiq (KSA) and three from Jebel Balas (KSA) are deposited in the WMLC and are *T.setosus*, but are not considered to be types. These specimens are from a locality not indicated in the original publication (Collingwood 1985). A Neotype for the species is herein designated to maintain the nomenclatural stability. Bolton (2007) already indicated that no type material of *T.setosus* could be located in NHMB or WMLC. He mentioned the presence of the two workers from Shaqiq (examined above) labelled as types, but with different locality data than the type material listed in the original description. Bolton (2007) concept of *T.setosus* was based on these two specimens.

#### Ecological notes.

Workers were collected from diverse habitats in the southwestern mountains of the KSA: Wadi Turabah (Al Bahah Province). A nest series was found under a rock next to an old *Acacia* (Fabaceae), where several workers were ascending the trunks and the twigs of these native plants, a foraging behavior mentioned by Bolton (2007). Several workers of Formicinae*Lepisiotaobtusa* (Emery 1901) were found foraging in the same area. This site is in a valley that has flowing drainages during the rainy season and supports a remarkable diversity of native vegetation that flourishes after the rains.

In Shohba Forest (Al Bahah Province) this species was found foraging on a trunk of *Acacia* sp. and next to a *Juniperusprocera* Hochst. exEndl. tree (Cupressaceae). In Wadi El Zaraeb (Al Bahah Province) workers of *T.setosus* were found under a rock near a *J.procera* tree in an area of scattered trees of OleaeuropeaL.subsp.africana (Mill.) PS Green (Oleaceae) and *Dodonaeaviscosa* Jacq. (Sapindaceae). In Beljorashi Forest (Al Bahah Province), this species was observed under an *Acacia* tree. In Al Sawda Mountains and in the Raydah Nature Preserve (Asir Province), workers of *T.setosus* were foraging on the ground where the soil was dry and rich in decaying organic material.

*Technomyrmexsetosus* was also collected from Wadi Al-Farah (Medina Province) (Collingwood and Agosti 1996), a mountainous rocky region with steep hillsides. The plant cover includes some *Acacia* trees, perennial bushes, and shrubs (Abo-Khatwa et al. 1980). The species was collected from Wadi Shuqub (Collingwood 1985), a site with dense *Balanitesaegyptiaca* (L.) Delile (Zygophyllaceae), perennial vegetation and *Acacia* woods (Büttiker 1981).

#### Geographic range.

*Technomyrmexsetosus* was originally described from KSA and has been recorded from Oman and Yemen (Collingwood and Agosti 1996) and is apparently an endemic species of the Arabian Peninsula.

### Technomyrmex vexatus

Taxon classificationAnimaliaHymenopteraFormicidae

(Santschi, 1919)

Figure 8A, B, C

Tapinoma vexatum Santschi 1919:220, Syntype male, Morocco: Tanger, 1897 (Vaucher), CASENT0911580, (NHMB), (examined), Palearctic.
Tapinoma (Tapinoptera) vexatum Santschi, 1925: 348. Combination in Technomyrmex by Cagniant and Espadaler 1993: 92.
Technomyrmex bruneipes : Collingwood and Agosti 1996; Fauna of Arabia 15: 361 [misidentification].

#### Description.

**Worker.** Measurements: TL: 3.1–3.4; HL: 0.72–0.78; HW: 0.68–0.76; SL: 0.64–0.70; PW: 0.44–0.48; WL: 0.90–0.96; Indices: CI: 94–99; SI: 90–94; OI: 22–25; EPI: 68–76; DTI: 118–130 (n=10, from Bolton 2007).

**Head.** Head with nearly straight posterior margin and convex sides; anterior clypeal margin feebly concave; eyes of moderate size with 10 ommatidia in the longest row (OI: 22–25), located just in front of the midlength of head, in full-face view outer margins of eyes just fail to protrude beyond sides of head. **Mesosoma.** Mesosonotum in profile with a flat anterior section that is slopping posteriorly and steeply to a well-developed narrow metanotal groove; propodeal dorsum short making a continuous curve with propodeal declivity. **Pilosity.** Head and mesosoma entirely lacking setae, first, second, and third gastral tergites without setae, fourth tergite with two or three pairs. **Sculpture.** Body finely and faintly microreticulate. **Colour.** Uniform dark brown, tarsi and funiculi paler yellow-bown.

#### Material examined.

Yemen, Sana’a, 15.3694°N, 44.1910°E, 2250 m, iii.1993, (Van Harten) (2 w, WMLC); Morocco, Septa, 29.v.1986, (X. Espadaler), det. B. Bolton, 2006, 1 w, CASENT0249804 (image examined).

#### Remarks.

*Technomyrmexvexatus* was originally described from Morocco. This species seems to exist as a series of isolated populations in rather restricted and specialized habitats throughout North Africa and eastward into the Arabian Peninsula, and perhaps Iran (B. Bolton, pers. comm.). Two species of the *T.gibbosus*-group are known from the Arabian Peninsula, the above record of *T.vexatus* from Yemen and *T.montaseri* from Oman. *Technomyrmexvexatus* was recorded for the first time from Palearctic (Gibraltar) by Guillem and Bensusan (2008).

#### Ecological and biological notes.

Nothing is known on ecology or biology of this species.

#### Geographic range

. Morocco (Santschi 1919, Cagniant and Espadaler 1993), Gibraltar (Guillem and Bensusan 2008). This species is newly recorded from Yemen and the Arabian Peninsula.

**Figure 8.**
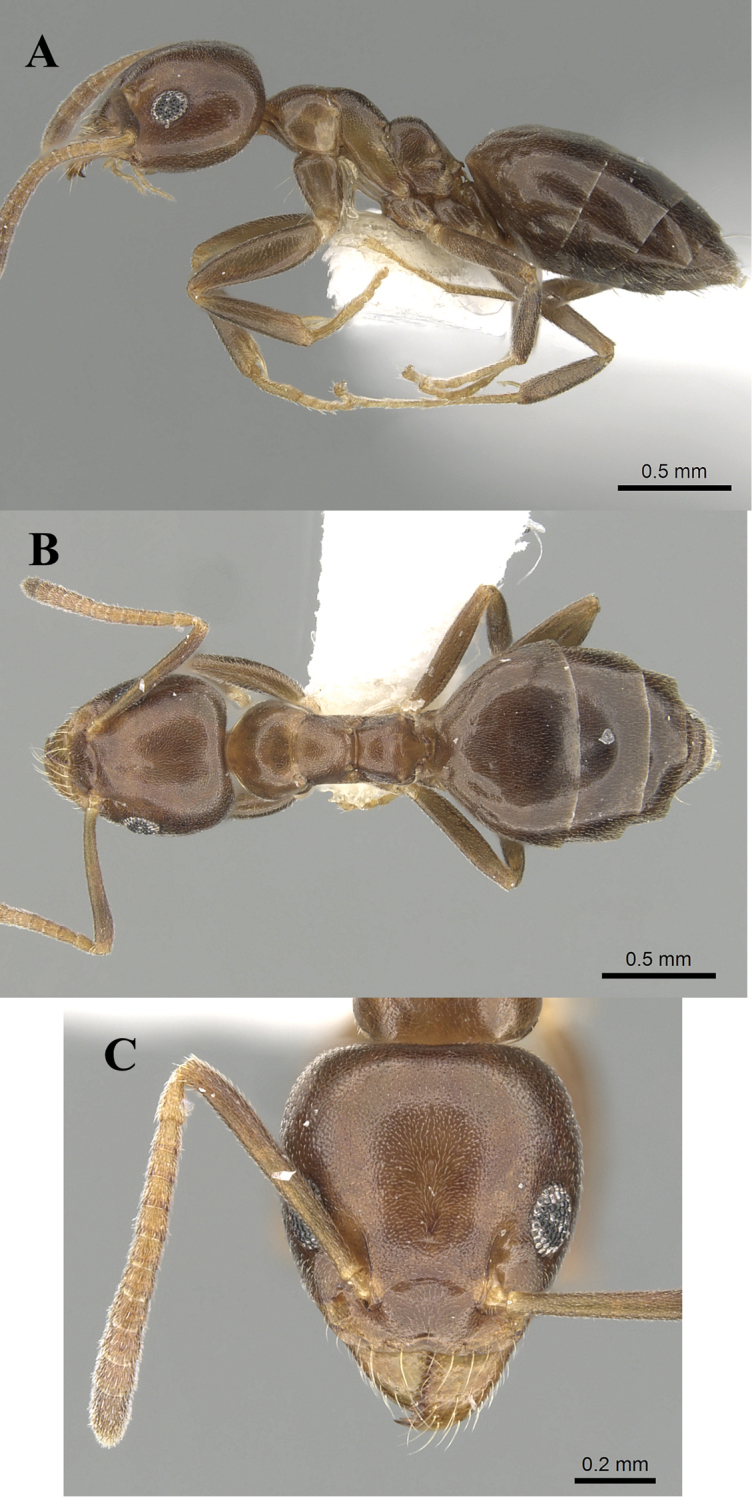
*Technomyrmexvexatus*, worker, CASENT0249804. **A** body in profile **B** body in dorsal view **C** head in full-face view, images from www.AntWeb.org.

### Material of unknown male of Technomyrmex

Taxon classificationAnimaliaHymenopteraFormicidae

sa01

Figure 9A, B, C, D

#### Measurements.

TL 3.25–3.75; HL 0.55–0.65; HW 0.50–0.67; SL 0.22–0.32; WL 1.25–1.40. Indices: SI 35–58; CI 81–112; EI 37–52 (n = 9).

#### Description.

**Head**. Head distinctly broader than long; mandible triangular, basal and masticatory margins with serrate denticles; apical tooth on masticatory margin longer than subapical one; scapes when laid back from their insertions just reach posterior margin of eyes; scape excluding its basal condyle shorter than length of funicular segments 1+2; first and second funicular segments cylindrical and straight; first funicular segment approximately one-third length of second; second and third funicular segments approximately twice as long as broad; third and fourth funicular segments straight; inner margin of eye entire, flat; anterior clypeal margin broad, convex, without a central notch or concavity of any type with five yellow setae, approximately as long as the maximum diameter of the scape; median portion of clypeus with a raised area which has curved anterior and posterior margins; anterior tentorial pit nearer antennal socket than mandibular insertion; anterolateral hypostoma reduced to a thin sclerite; medial hypostoma entire; palp formula 6, 4; third maxillary palp segment subequal in length to segment 4; third and fourth maxillary palp segments subequal; fifth approximately 2/3 × length of sixth. **Mesosoma.** Axillae medially compressed, anterior and posterior margins not parallel; anterior axillar suture concave; declivitous and dorsal faces of propodeum convex; dorsal face shorter than the declivitous face; propodeal angle indistinct. **Petiole.** Petiolar node strongly inclined anteriorly, its anterior margin much shorter than posterior margin in lateral view, not much expanded laterally; attachment to gaster narrow. **Genitalia**. Pygostyles present; apicoventral portion of basimere without projection. **Pilosity.** Whole body covered with pale appressed pubescence; mandibles with long yellow hairs. **Sculpture.** Body more or less shining with fine superficial microreticulation. **Colour.** Dull dark brown or black-brown.

#### Material examined.

KSA, **Asir Province**: Abha, Raydah Protectorate, 18.201583°N, 42.408933°E, 2600 m, 20.x.2014, (Al Dhafer et al.) (1 m); Saudi Arabia, Abha, Raydah Protectorate, 18.198067°N, 42.40725°E, 2600 m, 20.x.2014, (Al Dhafer et al.) (2 m); Saudi Arabia, Abha, Raydah Protectorate, 18.193633°N, 42.390333°E, 2600 m, 20.x.2014, (Al Dhafer et al.) (3 m); Abha, Raydah Protectorate, 18.198067°N, 42.40725°E, 2387 m, 20.x.2014, (Al Dhafer et al.) (1 m); Abha, Raydah Protectorate, 18.198067°N, 42.40725°E, 2387 m, 26.iv.2014, (Al Dhafer et al.) (1 m, CASENT0746638, CASC). **Al Baha Province**: Al Baha, Shada Al Ala, 19.8627°N, 41.301483°E, 1225 m, 3.vi.2014, (Al Dhafer et al.) (1 m); Saudi Arabia, Al Baha, Shada Al Ala, 19.842917°N, 41.311517°E, 1666 m, 3.vi.2014, (Al Dhafer et al.) (1 m); Saudi Arabia, Al Baha, Shada Al Ala, 19.842917°N, 41.311517°E, 1666 m, 27. i.2015, (Al Dhafer et al.) (1 m); all previous material was collected by e light trap and is deposited in KSMA.

#### Remarks.

Although there is no direct association between the male specimens studied here and the worker castes of *T.setosus*, it is highly likely that these male specimens are *T.setosus*. This supposition is supported by the relatively broad distribution of *T.setosus* in the southwestern mountains of the Arabian Peninsula and also with the scarcity and limited distribution of the closely related species, *T.briani*. This association may be confirmed with the use of the molecular techniques in the future.

**Figure 9.**
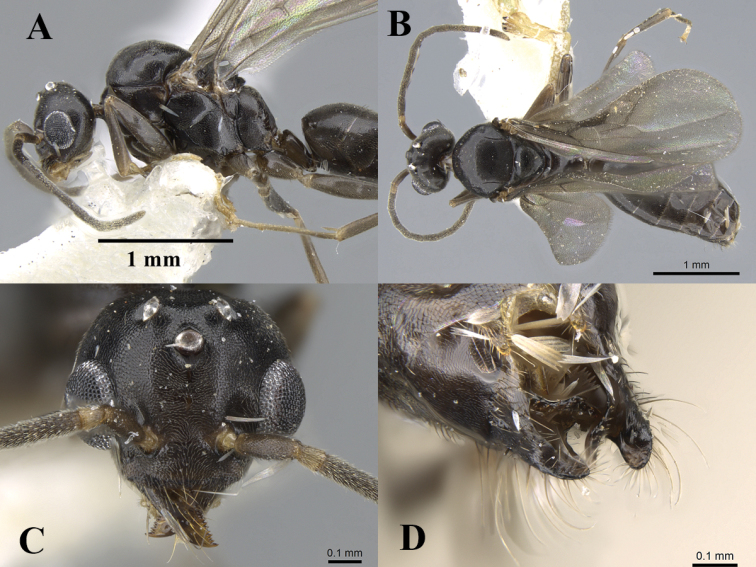
*Technomyrmex* sa01 male, CASENT0746638. **A** body in profile **B** body in dorsal view **C** head in full-face view **D** genitalia, images from www.AntWeb.org.

**Figure 10.**
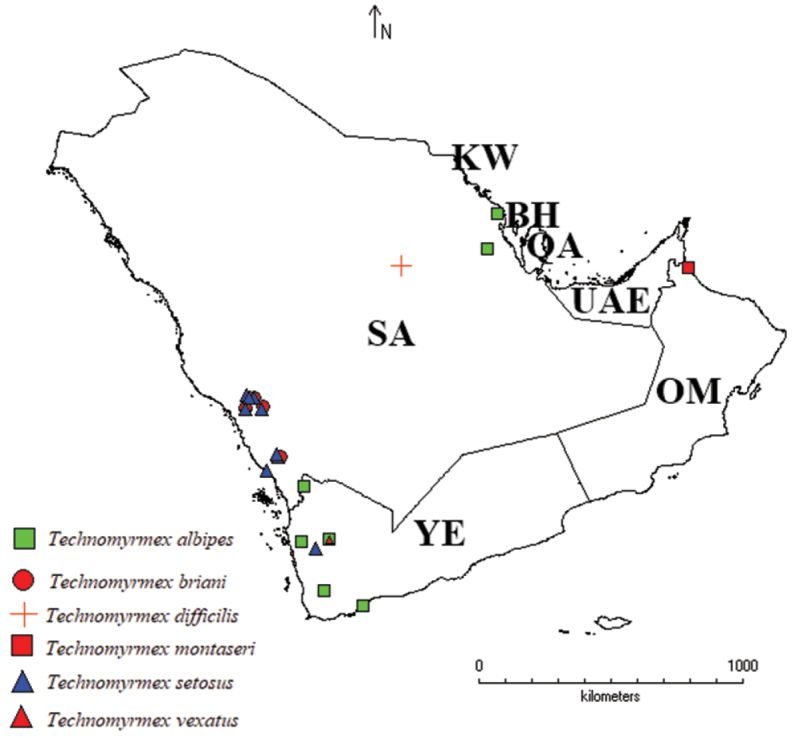
Distribution maps showing the known distribution ranges of *Technomyrmex* species on the Arabian Peninsula, BA (Bahrain), KW (Kuwait), OM (Oman), QA (Qatar), SA (Saudi Arabia), UAE (United Arab Emirates), and YE (Yemen).

## Discussion

The diversity of the genus *Technomyrmex* in the Arabian Peninsula is noticeably lower than in other ant genera. Bolton (2007) previous noted the lower abundance and species richness of the genus relative to the total ant fauna.

In terms of species endemism, *T.briani* and *T.setosus* apparently are restricted to the Arabian Peninsula. The genus is known to exhibit endemism in the Old World tropics (Fisher and Bolton 2016) and also in the Neotropics (Fernández and Guerrero 2008). The confined distribution of the Arabian *Technomyrmex* species in the southwestern region and the limited distribution in the eastern region may be due to the geographical separation by vast areas of deserts and the existence of the preferred habitats in the former region.

It is worth mentioning that relatively few specimens of *T.briani* are available as compared to *T.setosus*. Both species apparently have similar habitat preferences and geographical occurrence, despite equal efforts of collecting. It is apparent that both species prefers inhabiting grasslands of southwestern mountains of the KSA where *Acacia* and *J.procera* trees occur, and both prefer nesting under rocks at the elevated sites on both sides of valleys away from drainages.

## Supplementary Material

XML Treatment for Technomyrmex albipes

XML Treatment for Technomyrmex briani

XML Treatment for Technomyrmex difficilis

XML Treatment for Technomyrmex montaseri

XML Treatment for Technomyrmex setosus

XML Treatment for Technomyrmex vexatus

XML Treatment for Technomyrmex
